# Need for more robust research on the effectiveness of masks in preventing COVID-19 transmission

**DOI:** 10.2217/fvl-2021-0032

**Published:** 2022-04-19

**Authors:** Jingjing Nie, Linna Kang, Yaya Pian, Jihong Hu

**Affiliations:** ^1^National Center for Clinical Laboratories, Beijing Hospital, National Center of Gerontology; Institute of Geriatric Medicine, Chinese Academy of Medical Sciences, Beijing, 100730, China; ^2^Tianjin Binhai New Area Dagang Hospital, Tianjin, 300270, China

**Keywords:** COVID-19, mask, prevention, respiratory illness

## Abstract

The COVID-19 pandemic has caused dramatic death and infection worldwide, leading to a global public health crisis. As for precautions, scientists have different opinions on the effectiveness of masks in preventing COVID-19 transmission. Published studies suggested that medical masks may help in preventing respiratory virus infection. But the currently available experimental results are too preliminary to support an informed policy. In conclusion, we need more well-designed and robust research on the effectiveness of masks in preventing COVID-19 infection.

Confirmed and fatal cases of COVID-19 infection are still increasing around the world. Up to 1 February 2022, the COVID-19-confirmed cases have already surpassed more than 374 million, and deaths have exceeded 5.6 million. The daily new cases are still increasing in the USA, Brazil, India, Russia, England, etc. In China, outbreaks in clusters in local areas still exist during the winter, and the government and health administration have stayed alert to prevent new outbreaks. The COVID-19 pandemic has had a strong impact on society, the economy and daily life. Now we all realize that COVID-19 was not just a little flu and that preventing the spread of COVID-19 is still a very important task globally.

SARS-CoV-2 belongs to the group of respiratory-transmitted viruses. Respiratory viruses are transmitted via contact, droplets or aerosols [1]. Masks were one of the major protection measures at the beginning of the COVID-19 pandemic when vaccines or the antiviral drugs were unavailable. As for the mask, conflicting recommendations exist concerning whether we should use the mask to prevent respiratory illnesses, such as COVID-19 and SARS or not [2–6]. We need more robust research to provide evidence on the effectiveness of masks as one of the measures in preventing COVID-19 infection.

## Governments’ attitude toward wearing masks

The attitude toward wearing masks differs between governments. A preliminary comparison analysis showed that non-mask wearing countries (Spain, Italy, UK, Germany, France) had a significantly higher increase in COVID-19 cases than the mask-wearing countries (Japan, Thailand, China) at the time point of 2020 [7]. In China, governments recommended wearing masks from the beginning of the spread of COVID-19 to now. In the USA, the mask was required for air travel since 1 May 2020, and a federal law was implemented on 2 February 2021, that masks are required on planes, buses, trains and other forms of public transportation traveling into, within or out of the USA, and in transportation hubs such as airports and stations. But, at the beginning of COVID-19, the government did not recommend wearing masks in public. Now, USA CDC and the WHO recommend that people wear face masks in public or at gatherings, and tell people to wear a mask as a normal part of being around other people. However, both entities recommended just the opposite at an earlier time in the pandemic [8].

## Scientists have different opinions on wearing masks

By reviewing studies related to masks for the prevention of respiratory virus transmission, we found that scientists have different opinions on wearing masks [3,4]. Some scientists claimed that the masks are ineffective in preventing the spread of COVID-19. A perspective published in May 2020 in the ‘*New England Journal of Medicine*’ [3] concluded that cloth face masks offer little to no protection to the general population and healthcare workers. The author pointed out that with active COVID-19 patients, who may contaminate the environment, a mask will not protect healthcare workers or other people if they are not accompanied by hand hygiene, eye protection, gloves and a gown [3]. Schauer SG *et al.* [9] analyzed the effects of a county-wide mask order on COVID-19 mortality, ICU utilization and ventilator utilization in Bexar County, Texas, USA. There was no reduction in daily mortality, hospital bed, ICU bed or ventilator occupancy of COVID-19-positive patients after implementation of a mask order. A quantitative study evaluated the effectiveness of face masks in preventing airborne sneeze and cough droplets. The results showed that masks would not offer complete protection to a susceptible person from a viral infection in close (e.g., <6 ft) face-to-face or frontal human interactions, when the leakage percentages of these airborne droplets were expressed in terms of the number of virus particles [10]. Before the COVID-19 pandemic, a randomized trial that compares the efficacy of cloth masks to medical masks in hospital healthcare workers was conducted in 2015 [11]. A total of 1607 participants were randomized and divided into three groups: medical masks at all times on their work shift; cloth masks at all times on work shift and control group (standard practice, which may or may not include mask use). The results showed that the rates of all infection outcomes were significantly higher in the cloth masks group than in the medical mask group. Further, the penetration rate was almost 97 and 44% for cloth masks and medical masks, respectively. The study cautioned against the use of cloth masks and concluded that cloth masks should not be recommended for healthcare workers, particularly in high-risk situations.

Some studies claimed that face mask use was effective in preventing COVID-19. A systematic review and meta-analysis published in the journal ‘*Lancet*’ identified 44 relevant comparative studies in healthcare and non healthcare workers and concluded that both N95 and surgical masks might result in a sharp reduction in virus infection [4]. Another meta-analysis included 21 studies that suggested mask use provided a significant protective effect in preventing respiratory virus transmission [6]. A systematic review published in December 2020 in the ‘*American Journal of Infection Control’* compared the COVID-19 infection risk among participants who wore masks with those who did not [12]. Six case–controlled studies were conducted in four countries (China, the US, Thailand and Bangladesh). One of the studies involved participants from the general population, and the other five studies involved only healthcare workers. The study concluded that wearing a mask significantly reduced the risk of COVID-19 infection, especially for healthcare workers, which reduced the risk of infection by nearly 70 percent. A prospective cohort study [13] in the USA evaluated the potential risk for increased virus transmission by measuring the secondary attack rates (SARs) of COVID-19 between persons exposed when both parties were masked and those exposed when >1 person was unmasked. The analysis results suggested that proper mask use is very effective for reducing SARS-CoV-2 transmission, lowering the SAR among contacts by half. An epidemiologic study in Germany employed public regional data regarding the number of COVID-19 infections [5]. The study compared the increase of COVID-19 infection numbers in the region with the introduction of compulsory masks and regions without compulsory masks at different time points. The scientists found that the number of newly diagnosed COVID-19 infections decreased by 15–75% after 20 days of mandatory mask policy. By statistical analysis, the authors concluded that the daily growth rate of diagnosed COVID-19 infection would reduce by about 47% [5]. Another epidemiological study investigated the mortality caused by the SARS-CoV-2 in 200 countries from the beginning of the COVID-19 pandemic to 9 May 2020 [8]. The results showed that countries with the universal wearing of masks by the public have low levels of coronavirus mortality. In the discussion, the authors point out that Asian countries adopted widespread public mask usage strategy at the early period of the COVID-19 pandemic, and the coronavirus mortality was low in these countries, which at least tells us that masks seem unlikely to be harmful. The study concluded that the introduction of compulsory masks had led to fewer deaths than countries without mandatory masks [8].

Scientists explored the mechanisms of masks against droplet infections from a fluid physics point of view. Face masks can offer three fundamentally different kinds of protection [14]. The first fundamental protection mechanism was that the mask effectively prevents a smear infection, as the wearers of the masks no longer habitually touch the face and thus reduce the opportunity of bringing the virus from the hand into the mouth or nose. The second protection mechanism was that the flow resistance of the mask greatly limits the spread of viruses in the room. The third protection mechanism was that the inhalation of droplets containing viruses can be prevented by using a tight-fitting mask (N95) with particle filtering properties.

## Conclusion

From the published studies, one preliminary conclusion is that medical masks may help in preventing respiratory virus infection, and the effectiveness of cloth face masks needs further investigation [7,15,16]. As we all know, the currently available experimental results are too preliminary to support an informed policy. Most included trials in meta-analysis and reviews had poor design, reporting and sparse events. There is no RCT for the impact of masks on community transmission of COVID-19 until now due to logistical and ethical reasons (ethical issues prevent the availability of an unmasked control arm). The single epidemiologic study is not robust enough to back up a conclusion because of the population differences (age, gender, culture) and confounding factors (mask types, masks worn scientifically or not, vaccination and hand hygiene). We suggest that medical surgical masks and cloth face masks should be analyzed separately in future research. Using observational data to make causal inferences should be done very carefully. In addition, asymptomatic carriers should be considered a very important group in a community study. As the coronavirus vaccine has been marketed and administered in the general population in some countries, like China and the USA, it is worth investigating the necessity of wearing masks in vaccinated individuals.

In conclusion, we need more well-designed and robust research to study the effectiveness of masks as one of the strategies in preventing COVID-19 infection. Before we have enough evidence, governments should be careful in giving guidance to the public. It is suggested that informing the public that mask-wearing is one of the strategies that may help reduce spread, but the effectiveness of the mask needs more scientific evidence.

## Future perspective

With the accumulation of prevention and control experience with COVID-19 disease, determining the effectiveness of masks on preventing COVID-19 is worthwhile with the assessment based on previous data and robust randomized trials. It may also prove helpful to meet the challenge of the possible mutant viruses that appear in the future. As the coronavirus vaccine has been marketed and administered globally, whether the general population still needs to wear masks after mass vaccination is also worth investigating.

**Executive summary**

**Background**

- SARS-CoV-2 belongs to the respiratory transmitted virus group. Respiratory viruses are transmitted via contact, droplets or aerosols.
- Wearing a mask is one of the strategies to prevent the spread of respiratory transmitted viruses.

**Governments’ attitude toward wearing masks**

- The attitude toward wearing masks differs between governments.

**Scientists have different opinions on wearing masks**

- Scientists have different opinions on the effectiveness of masks in preventing COVID-19 transmission.
- Scientists have explored the mechanisms of masks against droplet infections from a fluid physics point of view.

**Conclusion**

- A preliminary conclusion is that medical masks may help in preventing respiratory virus infection. However, the currently available experimental results are too preliminary to support an informed policy.
- More well-designed and robust research on the effectiveness of masks in preventing COVID-19 infection is needed.

## References

[1] Kutter JS, Spronken MI, Fraaij PL, Fouchier RA, Herfst S. Transmission routes of respiratory viruses among humans. Curr. Opin. Virol. 28, 142–151 (2018). 29452994 10.1016/j.coviro.2018.01.001 PMC7102683

[2] Bundgaard H, Bundgaard JS, Raaschou-Pedersen DET Effectiveness of adding a mask recommendation to other public health measures to prevent SARS-CoV-2 infection in Danish mask wearers: a randomized controlled trial. Ann. Intern. Med. 174(3), 335–343 (2021). 33205991 10.7326/M20-6817 PMC7707213

[3] Klompas M, Morris CA, Sinclair J, Pearson M, Shenoy ES. Universal masking in hospitals in the COVID-19 era. N. Engl. J. Med. 382(21), e63 (2020). 32237672 10.1056/NEJMp2006372

[4] Chu DK, Akl EA, Duda S, Solo K, Yaacoub S, Schunemann HJ. Physical distancing face masks, and eye protection to prevent person-to-person transmission of SARS-CoV-2 and COVID-19: a systematic review and meta-analysis. Lancet 395(10242), 1973–1987 (2020). 32497510 10.1016/S0140-6736(20)31142-9 PMC7263814

[5] Mitze T, Kosfeld R, Rode J, Walde K. Face masks considerably reduce COVID-19 cases in Germany. Proc. Natl Acad. Sci. USA 117(51), 32293–32301 (2020). 33273115 10.1073/pnas.2015954117 PMC7768737

[6] Liang M, Gao L, Cheng C Efficacy of face mask in preventing respiratory virus transmission: a systematic review and meta-analysis. Travel. Med. Infect. Dis. 36, 101751 (2020). 32473312 10.1016/j.tmaid.2020.101751 PMC7253999

[7] Li T, Liu Y, Li M, Qian X, Dai SY. Mask or no mask for COVID-19: a public health and market study. PLoS ONE 15(8), e0237691 (2020). 32797067 10.1371/journal.pone.0237691 PMC7428176

[8] Leffler CT, Ing E, Lykins JD, Hogan MC, McKeown CA, Grzybowski A. Association of country-wide coronavirus mortality with demographics, testing, lockdowns, and public wearing of masks. Am. J. Trop. Med. Hyg. 103(6), 2400–2411 (2020). 33124541 10.4269/ajtmh.20-1015 PMC7695060

[9] Schauer SG, Naylor JF, April MD, Carius BM, Hudson IL. Analysis of the effects of COVID-19 mask mandates on hospital resource consumption and mortality at the county level. South Med. J. 114(9), 597–602 (2021). 34480194 10.14423/SMJ.0000000000001294 PMC8395971

[10] Akhtar J, Garcia AL, Saenz L, Kuravi S, Shu F, Kota K. Can face masks offer protection from airborne sneeze and cough droplets in close-up, face-to-face human interactions? A quantitative study. Phys. Fluids 32(12), 127112 (2020). 10.1063/5.0035072 PMC7757609 33362404

[11] MacIntyre CR, Seale H, Dung TC A cluster randomised trial of cloth masks compared with medical masks in healthcare workers. BMJ Open 5(4), e006577 (2015). 10.1136/bmjopen-2014-006577 PMC4420971 25903751

[12] Li Y, Liang M, Gao L Face masks to prevent transmission of COVID-19: a systematic review and meta-analysis. Am. J. Infect. Control 49(7), 900–906 (2021). 33347937 10.1016/j.ajic.2020.12.007 PMC7748970

[13] Riley J, Huntley JM, Miller JA, Slaichert ALB, Brown GD. Mask effectiveness for preventing secondary cases of COVID-19, Johnson County, Iowa, USA. Emerg. Infect. Dis. 28(1), 69–75 (2022). 34637377 10.3201/eid2801.211591 PMC8714203

[14] Kahler CJ, Hain R. Fundamental protective mechanisms of face masks against droplet infections. J. Aerosol. Sci. 148, 105617 (2020). 32834103 10.1016/j.jaerosci.2020.105617 PMC7321045

[15] Ataei M, Shirazi FM, Nakhaee S, Abdollahi M, Mehrpour O. Assessment of cloth masks ability to limit COVID-19 particles spread: a systematic review. Environ. Sci. Pollut. Res. Int. 29(2), 1645–1676 (2022). 34689269 10.1007/s11356-021-16847-2 PMC8541808

[16] Laxminarayan R, Wahl B, Dudala SR Epidemiology and transmission dynamics of COVID-19 in two Indian states. Science 370(6517), 691–697 (2020). 33154136 10.1126/science.abd7672 PMC7857399

